# Thyroid Hormone and Leptin in the Testis

**DOI:** 10.3389/fendo.2014.00198

**Published:** 2014-11-25

**Authors:** Cristiane Fonte Ramos, Ariane Zamoner

**Affiliations:** ^1^Laboratory of Morphometry, Metabolism and Cardiovascular Disease, Department of Anatomy, Biomedical Center, Institute of Biology, State University of Rio de Janeiro, Rio de Janeiro, Brazil; ^2^Departamento de Bioquímica, Centro de Ciências Biológicas, Universidade Federal de Santa Catarina, Florianópolis, Brazil

**Keywords:** leptin, thyroid hormones, male reproductive tissue, testis, adipose tissue

## Abstract

Leptin is primarily expressed in white adipose tissue; however, it is expressed in the hypothalamus and reproductive tissues as well. Leptin acts by activating the leptin receptors (Ob-Rs). Additionally, the regulation of several neuroendocrine and reproductive functions, including the inhibition of glucocorticoids and enhancement of thyroxine and sex hormone concentrations in human beings and mice are leptin functions. It has been suggested that thyroid hormones (TH) could directly regulate leptin expression. Additionally, hypothyroidism compromises the intracellular integration of leptin signaling specifically in the arcuate nucleus. Two TH receptor isoforms are expressed in the testis, TRa and TRb, with TRa being the predominant one that is present in all stages of development. The effects of TH involve the proliferation and differentiation of Sertoli and Leydig cells during development, spermatogenesis, and steroidogenesis. In this context, TH disorders are associated with sexual dysfunction. An endocrine and/or direct paracrine effect of leptin on the gonads inhibits testosterone production in Leydig cells. Further studies are necessary to clarify the effects of both hormones in the testis during hypothyroidism. The goal of this review is to highlight the current knowledge regarding leptin and TH in the testis.

## Introduction

The role of thyroid hormones (TH) and leptin in testicular physiology is not fully understood. Receptors for both hormones are present in the testis (1, 2), and some reproductive functions have been described as being a result of TH or leptin actions in the testis, although the interaction between these two hormones should be further investigated.

**Figure 1:**
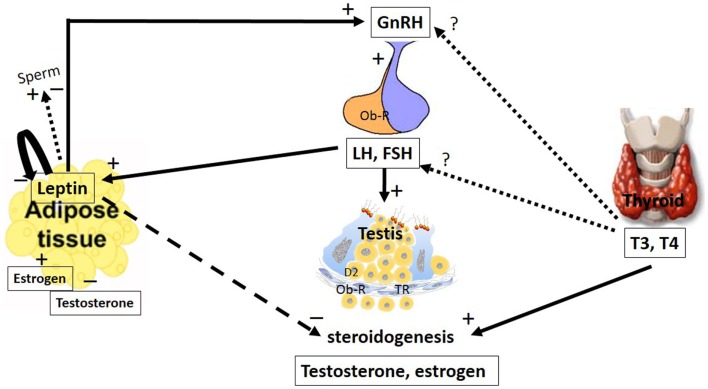
**The major pathways by which leptin and TH interact with the hypothalamic–pituitary axis to regulate testis function**. The hypothalamic–pituitary–gonadal axis controls reproduction. GnRH is secreted from the hypothalamus by GnRH expressing neurons, stimulating the anterior portion of the pituitary gland to produce and secrete LH and FSH, whose function is to stimulate the gonads to produce estrogen and testosterone. Leptin plays a role in reproduction by stimulating GnRH secretion. Stimulatory and inhibitory effects of leptin on sperm were described. Additionally, leptin has an inhibitory effect on the gonads, inhibiting steroidogenesis and decreasing serum levels of testosterone and estradiol. These hormones could regulate leptin secretion in a feedback mechanism; however, estradiol stimulates and testosterone inhibits leptin secretion. LH and FSH are other hormones that stimulate leptin secretion. Leptin is capable of autoregulating its own secretion. The role of the thyroid hormones, T_4_ and T_3_, at the hypothalamic and pituitary levels remain controversial. However, in the testes, these hormones stimulate steroidogenesis. The arrays indicate solid (stimulatory), dashed (inhibitory), and dotted (uncertain) signals. GnRH, gonadotropin-releasing hormone; LH, luteinizing hormone; FSH, follicle-stimulating hormone; T_3_, 3,5,3′-triiodo-l-thyronine; T_4_, thyroxine; Ob-R, leptin receptors; TR, thyroid receptor.

Leptin plays a key role in body weight homeostasis and has recently emerged as a relevant neuroendocrine mediator in different cell types, including testicular cells (3). This hormone appears to act by inhibiting testicular steroidogenesis, leading to reduced levels of testosterone and modulation of gene expression (Figure 1).

The involvement of TH in the modulation of male reproductive system development and function has been neglected for several years, because of the demonstration that the adult male gonad was metabolically unresponsive to these hormones in the 1950s (4). In addition, although hyper- and hypothyroidism have no apparent clinical relevance regarding signs and symptoms related to male gonadal function, compared with the systemic effects induced by these diseases, it has been demonstrated that thyroid dysfunction might affect biochemical, morphological, and physiological aspects of testicular development (4–8). In recent decades, several studies have reported important roles for TH in modulating testicular development (2, 9–11).

The TH effects involve the proliferation and differentiation of Sertoli and Leydig cells during development, spermatogenesis, and steroidogenesis, and its disorders are correlated with sexual dysfunction. An endocrine and/or direct paracrine effect of leptin on the gonads inhibits testosterone production in Leydig cells. Although further studies are necessary to clarify the effects of these hormones in the testis during hypothyroidism, the goal of this review is to highlight the current knowledge regarding leptin and TH in the testis.

## Leptin

### Synthesis and secretion

Adipose tissue is recognized as an endocrine organ that secretes steroid hormones, including glucocorticoids, growth factors, enzymes, and pro- and anti-inflammatory adipocytokines (12, 13). Leptin is the prototype adipokine that was identified as the product of the *ob* gene by its action in reducing appetite, increasing energy expenditure through action in the brain and then decreasing body weight and fat mass (14). Although anthropometric and clinical features (e.g., gender, fat mass/fat distribution, hormones, and cytokines) might influence the secretion pattern of leptin, the crucial factors that regulate serum leptin levels appear to be caloric intake and the amount of energy stored in adipocytes (15).

Leptin acts by activating leptin receptors (Ob-Rs). Several Ob-R isoforms, resulting from alternative splicing, convey differing biological activities and are involved in mediating the actions of leptin in the brain and peripheral organs. The long isoform (Ob-Rb) is expressed abundantly in the hypothalamic arcuate nucleus (ARH), ventromedial (VMN), and dorsomedial nuclei (DMN), and is the predominant signaling form of the receptor (16). Ob-Rb has the longest cytoplasmatic domain, which contains the Janus Kinase (JAK) binding domain, box 1 and 2, and the consensus sequence for the signal transducers and activators of transcription (STAT) binding. Other forms have no (Ob-Re) or short (Ob-Ra, Ob-Rc–Ob-Rf) cytoplasmatic domains, which contain only box 1 (17). These short isoforms of the leptin receptor are distributed in almost all the peripheral tissues, including ovary (18, 19), prostate (20–22), and testis (23) tissues, suggesting the direct effects of leptin on these organs. In addition to JAK/STAT, other pathways are involved in leptin signaling, such as mitogen-activated protein kinase (MAPK), including extracellular factor-regulated kinases 1 and 2 [ERK1 and ERK (2)] and 5′-AMP-activated protein kinase (AMPK). Additionally, leptin presents cross talk with insulin-induced pathways by stimulating insulin receptor substrates (IRS) and then initiating phosphoinositide 3-kinase activity (PI3K) (17, 24, 25). Leptin has been reported to induce suppressors of cytokine-signaling 3 (SOCS) expression, which are capable of inhibiting the JAK/STAT pathway via a feedback mechanism (26). During prolonged receptor stimulation by leptin, the inhibition of JAK phosphorylation is mediated by SOCS3, and leptin can act as a negative regulator of its own signaling.

**Figure 2:**
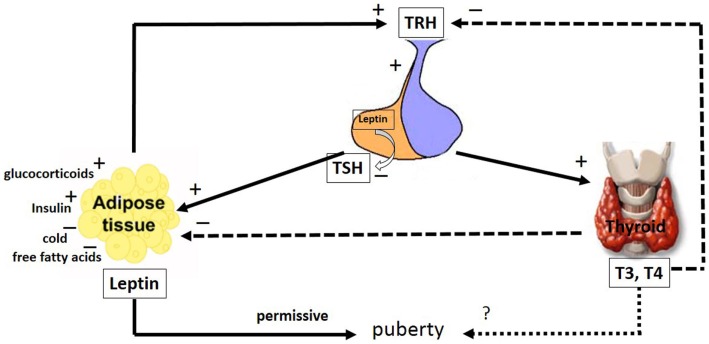
**The major pathways by which leptin interacts with the hypothalamic–pituitary–thyroid axis**. The hypophysiotropic TRH neurons are metabolic integrators that are regulated by leptin and the thyroid hormones. TRH is secreted from the PVN of the hypothalamus and stimulates the anterior portion of the pituitary gland to produce and secrete TSH, whose function is to stimulate the thyroid gland to produce the thyroid hormones, T_4_ and T_3_. These hormones regulate the activity of TRH neurons in the hypothalamus by a classical negative feedback pathway. Leptin stimulates TRH secretion in the hypothalamus. Leptin produced in the pituitary is capable of inhibiting TSH secretion. Leptin secretion from adipose tissues is regulated by stimulatory factors such as TSH, glucocorticoids, T_4_ and T_3_ and insulin as well as inhibitory factors such as, cold and free fatty acids. Leptin and the thyroid hormones, T_4_ and T_3_, have a role in puberty; however, whereas the leptin role is permissive, the T_4_ and T_3_ role remains uncertain. The arrays indicate: solid (stimulatory), dashed (inhibitory), dotted (uncertain) signals. TRH, thyreotrophin release hormone; TSH, pituitary tireotropin; T_3_, 3,5,3′-triiodo-l-thyronine; T_4_, thyroxine. See text for details.

Leptin secretion could be regulated by different mechanisms (Figure 2). In adipocyte tissue, leptin secretion could be stimulated by insulin, glucocorticoids, and cytokines (i.e., tumor necrosis factor a), whereas leptin release is inhibited by catecholamines, free fatty acids, cold exposure, and TH (27, 28). Estrogens induce leptin production, whereas androgens suppress it (Figure 1), thus explaining the sexual dimorphism in serum leptin levels (29, 30). A negative correlation between leptin and testosterone levels has been described in men and boys (31, 32). It has been demonstrated that leptin could control its own synthesis in adipose tissues by a negative feedback mechanism between the hormone and its receptor (Figure 1) (33). This autoregulatory mechanism was also shown in the prostate (34), however, there is no report of this mechanism in relation to the testis.

### Leptin and the hypothalamic–pituitary–gonadal axis

Leptin inhibits appetite and weight gain by its action in the hypothalamus by activating proopiomelanocortin (POMC)/cocaine- and amphetamine-related peptide (CART) while inhibiting the neuropeptide Y (NPY)/agouti-related protein (AgRP) neurons (35–38). In addition to this central action, leptin has been implicated in other roles, including reproductive ones where leptin stimulates gonadotropin-releasing hormone (GnRH) secretion (Figure 1). However, if leptin role is direct or not is still controverse. Some studies suggest a direct role of leptin based on the fact that the neurons secreting GnRH express Ob-R (39, 40). Watanobe (41) showed that leptin acts directly at the median eminence (ME), the anatomical structure where the axonal fibers of GnRH neurons are terminated before the neurohormone is released into the portal circulation (42); however, little or no co-expression of the leptin receptor is demonstrated at the medial preoptic area (MPOA) of the hypothalamus, which is the site where the majority of the GnRH neuronal cell bodies exist (42). This result, among others (43–45), suggests that leptin might influence GnRH secretion indirectly through interneurons and other pathways.

In the rat testis, leptin, at doses ranging from 2 to 500 ng/mL, exerted dose-dependent inhibitory effects on human chorionic gonadotropin (hCG)-stimulated testosterone production (46). Therefore, in addition to having a stimulatory effect at the hypothalamic–pituitary level, leptin appears to have an inhibitory effect at the gonadal level.

### Other sites that mediate leptin action on reproduction

Gonadotropin-releasing hormone is also regulated by adiponectin, which is an adipocytokine produced by adipose tissue and regulates metabolic function, as does leptin; the amount of GnRH in the circulation is inversely proportional to body fat (47). It has been demonstrated that adiponectin might provide a link between obesity and abnormal reproductive functioning by decreasing luteinizing hormone (LH) secretion and inhibiting GnRH receptor (GnRHR) mRNA expression in the pituitary (48, 49). At the level of the hypothalamus, adiponectin reduces GnRH expression and secretion (50), whereas GnRH decreases adiponectin expression in the pituitary (51). Additionally, adiponectin appears to mediate basal and hCG-stimulated testosterone secretion by the testis (52, 53) in a rat model.

Ghrelin is another orexigenic factor that acts with NPY and AgRP to influence feeding and reproduction. Whereas leptin stimulates GnRH secretion, ghrelin exerts a negative effect on this hormone secretion and on GnRH-induced gonadotropin secretion; however, it appears that at basal conditions, ghrelin presents stimulatory actions on LH and follicle-stimulating hormone (FSH) secretion (54). Relative to NPY, both stimulatory and inhibitory effects on GnRH have been observed (55–57). AgRP is expressed in leptin responsive neurons of the ARH and the ablation of such neurons in leptin or Ob-R deficient mice restores fertility (58–60).

Among the anorexigenic factors that affect the reproductive system, the α-melanocyte-stimulating hormone (α-MSH) from POMC neurons and CART from interneurons of the ARH mediate the leptin influence on GnRH secretion (61, 62). Additionally, nitric oxide (NO) released from adrenergic interneurons is capable of inducing GnRH release from GnRH neurons (23).

Another site that mediates leptin action on reproduction is the kisspeptin neurons in the ARH. Although a small amount of kisspeptin might be sufficient to trigger puberty (63), deletion of Ob-R from kisspeptin neurons (64) or ablation of more than 95% of kisspeptin neurons prior to puberty resulted in normal puberty and fertility (65). It appears that leptin signaling in these neurons arise only after completion of sexual maturation (66). Kisspeptin neurons connect to NPY and POMC neurons (67) are another link to the integration of leptin effects on nutrition and reproduction. Kisspeptin neurons have insulin receptors that could be interesting for their effect on reproduction. Deletion of the insulin receptor from kisspeptin neurons showed a puberty delay; however, fertility was normal (68), suggesting that insulin is the potential mediator of reproduction from kisspeptin neurons.

### Expression of leptin and Ob-R in testis

In 1997, the first demonstration of Ob-R expression in the murine testis was published. The authors showed the messenger RNA (mRNA) for the common extracellular domain of Ob-R in spermatic and Leydig cells by *in situ* hybridization (1). Later, several studies showed that it is ubiquitous among species. Ob-R specific immunostaining was observed in the testicular interstitium of rats at an embryonic age of 19.5 days and not at an embryonic age of 14.5 days, whereas in postnatal life, it was evident only after sexual maturation (35, 60, 90 days old) and was confined to Leydig cells. No immunoreaction was observed in the seminiferous tubules. Ob-R mRNA expression was present at all ages. Ob-Ra and Ob-Rb mRNA presented a similar expression pattern, increasing from younger to older ages. The authors demonstrated that the Ob-R mRNA levels were higher in late versus early embryonic testes, as well as in adult versus immature rat testes (69). Other studies have shown a developmental Ob-R mRNA expression from 15- to 75-day old rats in constant relative levels; however, if only Ob-Rb is considered, its expression was higher near puberty, at 30 days, declining after that period (70).

Leptin expression has been detected by immunohistochemistry techniques in mouse germ cells that are cell-type and stage specific and it was undetected in mouse Leydig cells. The authors suggest that leptin produced in testicular stem cells during the developmental stage might act on these cells in an autocrine manner to mediate their renewal (71). The developmental expression profiles of Ob-Ra, Ob-Rb, and Ob-Re show constant mRNA expression in 5-, 10-, 20-, 30-, and 60-day-old mice testis. However, in purified mouse Leydig cells, in which mRNA for leptin and Ob-Rb are not present in 14-and 60-day old mice, Ob-Ra and Ob-Re are expressed at both ages (71). Recently, mRNA for different Ob-R isoforms was detected in the adult rat testis (21).

Leptin caused STAT 3 phosphorylation after *in vitro* incubation of isolated seminiferous tubules, demonstrating that Ob-R is functional and an activator of signal transduction mechanisms in germ cells (72). Corroborating this result, recently it was shown that leptin activates STAT3 signaling in the testis and that SOCS3 expression is regulated by leptin in this tissue (73).

### Leptin actions

Considering that impaired reproductive function in the genetically obese (ob/ob) mouse was previously demonstrated (74, 75), leptin might be one of the hormones involved in the pathogenesis of infertility. Corroborating this hypothesis, the exogenous intraperitoneal administration of leptin in sterile ob/ob mice eliminated the sterility defect (76).

In the testis, it has been demonstrated that leptin might pass the blood–testis barrier by a passive, non-saturable process (77, 78). In this tissue, leptin acts as an inhibitory signal for testicular steroidogenesis (Figure 1), which might partially explain the link between decreased testosterone secretion and hyperleptinemia in obese men (79, 80). Decreased gene expression of some upstream factors in the steroidogenic pathway, such as steroidogenic factor-1 (SF-1), steroidogenic acute regulatory protein (StAR), and cytochrome P450, has been demonstrated to be involved in the molecular mechanism responsible for leptin-induced inhibition of testosterone secretion (81). Moreover, the inhibitory effects of leptin on steroidogenesis have been observed in the adrenal gland and ovaries, which are steroidogenic tissues as well (82–84).

Besides testosterone synthesis, leptin can also regulate estrogen synthesis by regulating aromatase enzyme in other tissues of the reproductive system such as prostate and mammary gland (20, 34, 85, 86). We were unable to find studies showing the effects of leptin upon aromatase in the testis.

Some other effects of leptin that can affect fertility have been already demonstrated. Leptin can alter the weight and volume of the testicles, the diameter of the seminiferous tubules, and the numbers of spermatogonia, spermatocytes, sperm, and Leydig cells (73). Also the fact that leptin is present in the human seminal plasma (87–89) and Ob-R is present at the tail region of ejaculated spermatozoa (89) as well as the relationship between leptin and sexual hormones suggests that leptin could directly or indirectly regulate sperm function.

### Obesity and testis

The effects of obesity on testis and male reproduction have been demonstrated in recent years (90). The decrease in testosterone levels as a consequence of the high leptin levels present in obese patients (91) might cause sexual dysfunction (92). However, the mechanisms involved in the association between obesity and a decrease in male fertility are complex and not completely understood on the basis of the current literature.

A high-fat diet leads to increased leptin levels, decreased plasma testosterone level, a decline in sperm motility, Leydig cell damage, and oxidative stress, as well as a decreased testis and epididymis relative coefficient in mice. The reproductive hormone imbalance observed in obesity might affect the antioxidant status in testes. The authors suggest that the excessive oxidative stress induced by obesity affects the normal histological structures and function of testicular tissue (93). In addition, the association of testicular oxidative stress and alterations in male reproduction and fertility has been demonstrated in other conditions such as thyroid dysfunctions (94–97).

Although consensus on the effects of obesity on fertility exists, particularly in relation to sperm count, concentration and motility, there is not a complete overall agreement (98). The lack of consensus is possibly because the majority of studies involve men presenting to fertility clinics, which could clearly bias toward subfertile men, and many might have other lifestyle related co-morbidities affecting spermatogenesis. Different methodologies applied in various studies could contribute to the variability of the results. Some of the fertility alterations could occur because of the effects of leptin on other tissues of the reproductive systems that express Ob-R as well, such as the epididymis (99), prostate (20–22), and seminal vesicles (22).

Testosterone is a very important hormone for maintaining reproductive function and fertility. All the reproductive tissues and spermatogenesis are testosterone-dependent. Although androgen could regulate leptin synthesis and secretion in adipose tissues (29, 30) and leptin could regulate the expression of androgen receptors in others tissues, such as the prostate (20, 34), we were unable to find studies showing any interaction between leptin and testosterone or androgen receptors in the testis.

These findings clearly demonstrate that leptin functionally regulates the male gonadal axis by acting at different levels of the hypothalamic–pituitary–testicular system.

## Thyroid Hormone

### Thyroid hormone regulation

As major regulators of serum TH levels, the hypophysiotropic thyreotrophin release hormone (TRH) neurons of the paraventricular nuclei (PVN) play important roles in the control of energy homeostasis (100). These neurons regulate TH production through the release of TRH at the ME, which stimulates pituitary tireotropin (TSH) release (Figure 2). TSH then stimulates the thyroid gland to produce TH. These hormones regulate the activity of TRH neurons in the hypothalamus by a classical negative feedback pathway (Figure 2) (101–103). TRH neurons receive neuronal projections from POMC and NPY containing hypothalamic neurons (104–108), brainstem catecholaminergic neurons (103, 109, 110), and neurochemically uncharacterized neurons in the DMN (111).

Thyroid hormones play critical roles in normal pre- and postnatal growth and development. These hormones are essential for the regulation of metabolism in nearly all mammalian tissues, including the testis (112, 113).

### Deiodination

Proper intracellular TH concentrations are required for most hormonal actions and for cellular metabolism. Thyroxine (T_4_) is the major TH secreted from the thyroid gland; however, in target tissues other hormones could be produced. In peripheral tissues, there are important seleno enzymes known as deiodinases (D1, D2, and D3) that metabolize TH, regulating local TH availability (114). D1 and D2 catalyze the removal of the iodine atom in the outer ring position, generating 3,5,3′-triiodo-l-thyronine (T_3_). D3 catalyzes the removal of iodine from the inner ring position, generating an inactive hormone, as reverse T_3_ (3,3′,5′-triiodothyronine or rT_3_).

These three deiodinases, D1, D2, and D3, are expressed in the testis at different levels, from weanling to adult life (115). D3 activity predominates in the developmental period and then declines in adult life. Although D1 and D2 are present in the testis, their relative levels of activity indicate that D2 is the predominant activating enzyme in the testis (115, 116).

Thyroid hormone transport across the plasma membrane is a crucial step in TH signaling. Several TH transporter families have been identified; however, only specific TH cell-membrane transporter (MCT) 8 and 10 and organic anion-transporting polypeptide 1c1 (Oatp1c1) have been shown to be specific TH transporters (117). Inside the cell, T_3_ binds to nuclear THs, which are ligand-regulated transcription factors that bind THs and DNA enhancer sequences in the promoter region of target genes and interact with co-repressor and co-activator complexes (118, 119).

### Signaling

Thyroid hormone nuclear effects are mediated by TRa and TRb, which are members of the steroid/thyroid hormone receptor superfamily (120). The activation of these receptors modulates gene transcription and signal transduction to initiate intra-nuclear changes in cell metabolism (121, 122). Because the genomic actions of TH on gene regulation require many steps, TH-induced changes in gene expression are generally long lasting (113, 120).

The TR are encoded by two different genes, TRa and TRb. Alternative splicing leads to the production of several peptide isoforms, five of which have been described: TRa1, TRa2, TRa3, TRb1, and TRb2. TRa2 and TRa3 lack a hormone-binding domain and are hypothesized to function as TH inhibitors by competing for binding at the thyroid response elements (TREs), resulting in the suppression of transcription (123–128). Within the seminiferous epithelium, the expression of TRs is developmentally regulated, and Sertoli cells express the TRa1 and TRa2 isoforms (85).

The active TRa1 isoform is expressed in human and rat testicular Sertoli cells, with maximal expression in late fetal and early neonatal life. TRb1 is found in interstitial and germ cells during neonatal development as well as in the adult testis, although at a much lower level (129–132). The fetal and prepubertal ages are periods of maximal expression of TRa1 and TRa2 in Sertoli cells, demonstrating a critical window for TH action in the testis, in rats (2) and in human beings (133). The immature stage of sexual development coincides with the maximal TRa1 activity in response to T_3_ (2). The TRa2 isoform does not bind to T_3_, and it has been suggested that TRa2 most likely exerts a dominant negative effect on the actions of other TR isoforms (134). Considering the developmental regulation of TR expression in the Sertoli cells, it was shown that the ratio of TRa2/TRa1 increases progressively from the fetal period to adulthood. This increase reinforces the participation of TRa1 during the prepubertal period and, consequently, its involvement in testis differentiation and development (133). It could be proposed that TH play pivotal roles in testicular differentiation with maximal effects during the prepubertal period.

Although the classical mechanism of action for TH involves the modulation of gene transcription (genomic action), it has been frequently proposed that the membrane-initiated effects (non-genomic actions) of these hormones might be mediated by plasma membrane receptors. The effects triggered by cell-surface receptors are typically independent of protein synthesis (112). Although T_3_, T_4_, and rT_3_ might trigger non-genomic effects, T_4_ and rT_3_ are more potent than T_3_ in testicular cells (135–138), reinforcing a role for these hormones as physiological signals controlling reproductive functions through cell surface-initiated mechanisms.

### Thyroid hormone function in testis

T_3_ regulates the maturation and growth of the testis, controlling Sertoli and Leydig cell proliferation and differentiation during testicular development in rats and other mammalian species (5, 139). Possible mechanisms underlying the effects of TH on Sertoli cell proliferation have been recently proposed. The authors demonstrated that TH limits postnatal Sertoli cell proliferation by activation of the TRa1 present in these cells. They provided evidence that the regulation of the Cdk4/JunD/c-myc pathway might be involved in this negative control in mice (140). The testis size and sperm production are directly associated with the total Sertoli cell number during adulthood (141). The hormonal factors controlling the duration of Sertoli cell proliferation are critical determinants of male fertility (4). Although an adequate number of these cells is crucial for future male fertility, the factors controlling Sertoli cell survival, proliferation, and maturation require further investigation.

The effects of TH on Sertoli cells occurs through different and perhaps interconnected mechanisms (142) including the following: the expression of cell cycle regulators p27 and p21 (143); the inhibition of aromatase at the basal conditions and after FSH stimulus (144, 145) as well as the aromatase gene transcription (146); the increase in the levels of androgen-binding proteins (ABP) (129), which increases the availability of this steroid hormone (147, 148); the increase in the androgen receptor (AR) expression (129); the increase in the LH receptor (LHR) protein expression, although the mRNA is reduced (149); and the increase in the levels of the gap junction protein connexin 43 in the testis (150). For revision, see Tarulli et al. (142).

The Sertoli cells provide physical support to germ cells and are essential for the creation of an adequate and protected microenvironment for germ cell development. These cells are responsible for the secretion of a Cl^–^ and K^+^-rich fluid into the seminiferous tubule lumen (151, 152). The Ca^2+^-dependent Cl^−^ secretion by Sertoli cells into the extracellular fluid is under the control of extracellular nucleotide levels (153); extracellular ATP has been shown to induce intracellular Ca^2+^uptake in Sertoli cells (154). Our group previously demonstrated that T_3_ and T_4_ could inhibit NTPDase activity, with a consequent decrease in extracellular ATP hydrolysis; this reduction in hydrolysis leads to increased levels of ATP in the extracellular medium (10). It has been demonstrated that TH induces Ca^2+^-dependent signaling pathways in testicular cells (11, 112, 136, 155). It is possible to suggest that TH action might increase the extracellular ATP levels, leading to a Ca^2+^ influx and consequent chloride-rich fluid secretion by the Sertoli cells.

The expression of specific TRs in prepubertal Sertoli cells implies the existence of an early and critical influence of TH on testicular development and function (2). Although hypothyroidism leads to a marked delay in sexual maturation and development (5, 9, 96), hyperthyroidism accelerates the appearance of the seminiferous tubule lumen (97), which marks the maturation of the Sertoli cells (156). Delayed testicular cell maturation provoked by neonatal hypothyroidism (96) is associated with reduced serum levels of the gonadotropins, FSH and LH (6).

Modifications in Sertoli cell maturation could be associated with alterations in androgen metabolism. The androgen/estrogen balance is crucial for testis development and male reproduction. The maximal expression and activity of aromatase is observed in Sertoli cells during the prepubertal period (157, 158), the period coincident with the maximal response of these cells to TH (4). Alterations in aromatase activity and expression, as well as in estrogen receptor levels, were demonstrated to be under TH control (145, 159, 160). These estrogen-mediated events might affect androgen metabolism.

Thyroidectomy in adult rats led to decreased secretion of testosterone and 17*b*-hydroxy steroid dehydrogenase (HSD) activity (161). Leydig cells cultured from adult rats show an increase in testosterone and estrogen secretion after treatment with T_3_, under basal conditions and in response to LH (148). When Leydig cells are chronically stimulated with T_3_, there is an increase in the mRNA levels of the cytochrome P450 side-chain cleavage enzyme and a decrease in the cytochrome P450 17*a*-hydroxylase and 3*b*-HSD (162). An increase in the StAR mRNA and protein is observed after treatment of Leydig cells with T_3_, which contributes to steroid production (162, 163) (Figure 2).

Despite the important roles that THs play in the reproduction by acting in the testis, there is no consensus about how TH controls GnRH and gonandotropin synthesis and secretion. No pattern in the circulating gonadotropin hormones has been reported in hypothyroid conditions. These hormones could be unaltered, reduced, increased or LH reduced with unaltered FSH; and FSH could be increased with normal LH (147). In a recent study (149), the authors showed that rats submitted to hypothyroidism showed a reduction of the pituitary content of LH, although they presented a high level of LH in serum. They attributed this divergent result to impaired LH renal clearance, because hypothyroidism could alter clearance (164) of adrenal drug excretion (165). There was a marked decrease of total testosterone serum concentration and a reduction in the amount of testicular LHR, although the LH mRNA receptor expression was shown to be increased. Exposure of testicular cells to a high concentration of LH reduces LHR mRNA and protein (166), which could explain the results observed by Romano et al. (149). Another possibility would be the reduction of the testosterone negative feedback regulating LH and FSH.

### Interaction between leptin and thyroid hormones

The hypophysiotropic TRH neurons are metabolic integrators, fixing the set point of the hypothalamic–pituitary–thyroid axis. Several studies have demonstrated that leptin modulates thyroid function, acting in the hypothalamus, pituitary and thyroid. Additionally, leptin modulates the activity of deiodinase enzymes (167–171). This hormone stimulates the hypothalamic production of TRH directly at the PVN (106, 172–174) and indirectly via the ARH, which act as energy sensors. At the ARH, leptin upregulates the activity of the α-MSH neurons and down-regulates the activity of the NPY/AgRP neurons. The α-MSH neurons and NPY/AgRP neurons have stimulatory and inhibitory projections, respectively, to the TRH neurons (100, 106, 174–178).

Systemic administration of leptin increased the serum TSH concentration in rats, potentially because of the leptin action at the hypothalamus (Figure 2). Because the direct pituitary effect of leptin on TSH release was inhibitory, this TSH release might result from an autocrine–paracrine effect exerted by locally produced leptin (Figure 2) (170, 171). TSH significantly stimulated the leptin secretion by human adipose tissue *in vitro*, suggesting a new mechanism in the inter-relationship between adipose tissue and the thyroid axis (Figure 2) (179). It has been demonstrated that food deprivation, associated with low leptin levels, leads to decreased TSH synthesis in the pituitary and TRH in the hypothalamus (171, 180). The mechanisms underlying the secretion of tropic hormones from pituitary cells might involve the modulation of NO levels. Coiro and colleagues suggested that TRH stimulates TSH release via NO in humans (181). Corroborating these findings, Radwanska and Kosior-Korzecka (182) recently demonstrated that leptin-stimulated TSH secretion is dependent on NO release from the anterior pituitary cells of ewe lambs. In this context, the effect of leptin on the thyroid axis might modulate the effects of THs on the male reproductive system.

It has been proposed that leptin might be a trigger for the onset of puberty in children, because leptin levels increase in both girls and boys prior to pubertal gonadal activation (183). In girls, however, leptin concentrations continue to rise, likely due to stimulatory the effects of estrogen, while leptin concentrations decrease in boys, due to the inhibitory effects of testosterone (29, 30, 183, 184). However, data of Mann and colleagues (185) do not support the concept that a transient rise in leptin triggers the onset of puberty. They demonstrated that circulating leptin levels decline throughout puberty, and there is no transient rise of leptin prior or in association with the onset of puberty in male monkeys. Leptin, however, is currently thought to have a more permissive role in pubertal maturation, as the administration of exogenous leptin alone could not trigger early puberty in patients with congenital leptin deficiency (186). Although leptin probably is not the primary factor for puberty initiation, lower levels of this hormone could be associated with a delay in the onset of puberty (187). It has been shown that some developmental changes in the T_4_ levels parallel those of leptin. Whether the peripubertal changes observed in T_4_ levels suggest thyroid involvement in initiating pubertal events remains to be elucidated.

The relationship between TH and leptin serum levels is not completely understood; however, it has been demonstrated that leptin levels might be increased in the hyperthyroid state in humans (188). In addition, Asami and coworkers (189) have demonstrated a relationship between the serum levels of leptin and TH in children. A direct effect of leptin stimulating hepatic TH activation by modulation of deiodinase activity and expression was shown in chicken embryo hepatocytes (190). The authors demonstrated that the expression and activity of D1 were increased, whereas those of D3 were decreased, in leptin-treated cells. A D1-increased gene expression and activity in the adipose tissues of obese humans was doccumented, suggesting a role for T_3_, formed from T_4_ by D1 activation in response to leptin, in the modulation of adipose tissue metabolism (191). These studies support that TH and leptin might act in conjuction in the modulation of several cell functions.

Corroborating the relationship between TH and leptin, it has been shown that hypothyroidism reduces the expression of members of the Ob-Rb–STAT3 signaling pathway in the basomedial hypothalamus and pituitary of rats; in addition, hypothyroid rats are resistant to the acute anorectic action of leptin (192). Additionally, hypothyroid mice exhibited decreased circulating leptin levels because of a decrease in the fat mass and reduced leptin expression in white adipose tissue. In neurons of the ARH, hypothyroid mice showed increased leptin receptor Ob-R expression and decreased suppression of the cytokine-signaling 3 transcript levels (193).

The main pathways by which leptin and TH interact with the hypothalamic–pituitary axis to regulate testis function are summarized in Figures 1 and 2. This review is a first demonstration proposing explanations for the possible relationship between TH and leptin activity in the testis. Further studies are needed to clarify how these two important hormones might act together in modulating male reproduction.

## Conflict of Interest Statement

The authors declare that the research was conducted in the absence of any commercial or financial relationships that could be construed as a potential conflict of interest.

## References

[1] Hoggard N Mercer JG Rayner DV Moar K Trayhurn P Williams LM. Localization of leptin receptor mRNA splice variants in murine peripheral tissues by RT-PCR and in situ hybridization. Biochem Biophys Res Commun (1997) 232(2):383–7. 10.1006/bbrc.1997.6245 9125186

[2] Jannini EA Carosa E Rucci N Screponi E D’Armiento M. Ontogeny and regulation of variant thyroid hormone receptor isoforms in developing rat testis. J Endocrinol Invest (1999) 22(11):843–8. 10.1007/BF03343656 10710271

[3] Tena-Sempere M Barreiro ML. Leptin in male reproduction: the testis paradigm. Mol Cell Endocrinol (2002) 188(1–2):9–13. 10.1016/S0303-7207(02)00008-4 11911940

[4] Jannini EA Ulisse S D’Armiento M Thyroid hormone and male gonadal function. Endocr Rev (1995) 16(4):443–59 10.1210/er.16.4.443 8521789

[5] Holsberger DR Cooke PS. Understanding the role of thyroid hormone in Sertoli cell development: a mechanistic hypothesis. Cell Tissue Res (2005) 322(1):133–40. 10.1007/s00441-005-1082-z 15856309

[6] Palmero S de Marchis M Gallo G Fugassa E. Thyroid hormone affects the development of Sertoli cell function in the rat. J Endocrinol (1989) 123(1):105–11. 10.1677/joe.0.1230105 2572663

[7] Zamoner A Barreto KP Filho DW Sell F Woehl VM Guma FC Propylthiouracil-induced congenital hypothyroidism upregulates vimentin phosphorylation and depletes antioxidant defenses in immature rat testis. J Mol Endocrinol (2008) 40(3):125–35. 10.1677/JME-07-0089 18316471

[8] Zamoner A Barreto KP Filho DW Sell F Woehl VM Guma FC Hyperthyroidism in the developing rat testis is associated with oxidative stress and hyperphosphorylated vimentin accumulation. Mol Cell Endocrinol (2007) 267(1–2):116–26. 10.1016/j.mce.2007.01.005 17306450

[9] Holsberger DR Kiesewetter SE Cooke PS. Regulation of neonatal Sertoli cell development by thyroid hormone receptor alpha1. Biol Reprod (2005) 73(3):396–403. 10.1095/biolreprod.105.041426 15858214

[10] Zamoner A Bruno AN Casali EA Corbelini PF Diniz GP Barreto-Chaves ML Genomic-independent action of thyroid hormones on NTPDase activities in Sertoli cell cultures from congenital hypothyroid rats. Life Sci (2006) 80(1):51–8. 10.1016/j.lfs.2006.08.020 16978660

[11] Zamoner A Corbelini PF Funchal C Menegaz D Silva FR Pessoa-Pureur R. Involvement of calcium-dependent mechanisms in T3-induced phosphorylation of vimentin of immature rat testis. Life Sci (2005) 77(26):3321–35. 10.1016/j.lfs.2005.05.042 15985269

[12] Laclaustra M Corella D Ordovas JM Metabolic syndrome pathophysiology: the role of adipose tissue. Nutr Metab Cardiovasc Dis (2007) 17(2):125–39 10.1016/j.numecd.2006.10.005 17270403

[13] Matsuzawa Y. The metabolic syndrome and adipocytokines. FEBS Lett (2006) 580(12):2917–21. 10.1016/j.febslet.2006.04.028 16674947

[14] Halaas JL Gajiwala KS Maffei M Cohen SL Chait BT Rabinowitz D Weight-reducing effects of the plasma protein encoded by the obese gene. Science (1995) 269(5223):543–6 10.1126/science.7624777 7624777

[15] Kelesidis T Mantzoros CS. The emerging role of leptin in humans. Pediat Endocrinol Rev. (2006) 3(3):239–48. 16639389

[16] Héritier A Charnay Y Aubert ML Regional distribution of mRNA encoding the long form of leptin receptor in the mouse brain. Neurosci Res Commun (1997) 21(2):113–8 10.1002/(SICI)1520-6769(199709/10)21:2<113::AID-NRC214>3.3.CO;2-X

[17] Hegyi K Fulop K Kovacs K Toth S Falus A Leptin-induced signal transduction pathways. Cell Biol Int (2004) 28(3):159–69 10.1016/j.cellbi.2003.12.003 14984741

[18] da Silveira Cavalcante F Gombar FM Ferreira RV da Silva Faria T Costa WS Sampaio FJ Maternal protein-energy and energy-restricted diets during lactation possibly could program folliculogenesis and the ovarian expression of leptin and its different isoform receptors in rats. Fertil Steril (2009) 92(5):1755–7 10.1016/j.fertnstert.2009.05.037 19591986

[19] Ryan NK Van der Hoek KH Robertson SA Norman RJ. Leptin and leptin receptor expression in the rat ovary. Endocrinology (2003) 144(11):5006–13. 10.1210/en.2003-0584 12959975

[20] Alves-Pereira JL Colli S Marques DS Sampaio FJ Ramos CF. Molecular and morphometric analysis of the rat ventral prostate injected with leptin. Regul Pept (2012) 176(1–3):6–12. 10.1016/j.regpep.2012.02.002 22387703

[21] Gombar FM Ramos CF. Perinatal malnutrition programs gene expression of leptin receptors isoforms in testis and prostate of adult rats. Regul Pept (2013) 184:115–20. 10.1016/j.regpep.2013.03.009 23499808

[22] Malendowicz W Rucinski M Macchi C Spinazzi R Ziolkowska A Nussdorfer GG Leptin and leptin receptors in the prostate and seminal vesicles of the adult rat. Int J Mol Med (2006) 18(4):615–8. 10.3892/ijmm.18.4.615 16964413

[23] Yu WH Walczewska A Karanth S McCann SM. Nitric oxide mediates leptin-induced luteinizing hormone-releasing hormone (LHRH) and LHRH and leptin-induced LH release from the pituitary gland. Endocrinology (1997) 138(11):5055–8. 10.1210/endo.138.11.5649 9348239

[24] Lin HY Yang SH Tang HY Cheng GY Davis PJ Grasso P. Biologically active leptin-related synthetic peptides activate STAT3 via phosphorylation of ERK1/2 and PI-3K. Peptides (2014) 57:95–100. 10.1016/j.peptides.2014.04.007 24819473

[25] Elias CF Purohit D. Leptin signaling and circuits in puberty and fertility. Cell Mol Life Sci (2013) 70(5):841–62. 10.1007/s00018-012-1095-1 22851226 PMC3568469

[26] Sahu A. Leptin signaling in the hypothalamus: emphasis on energy homeostasis and leptin resistance. Front Neuroendocrinol (2003) 24(4):225–53. 10.1016/j.yfrne.2003.10.001 14726256

[27] Ahima RS. Adipose tissue as an endocrine organ. Obesity (2006) 14(Suppl 5):242S–9S. 10.1038/oby.2006.317 17021375

[28] Considine RV Regulation of leptin production. Rev Endocr Metab Disord (2001) 2(4):357–63 10.1023/A:1011896331159 11725722

[29] Machinal F Dieudonne MN Leneveu MC Pecquery R Giudicelli Y. In vivo and in vitro ob gene expression and leptin secretion in rat adipocytes: evidence for a regional specific regulation by sex steroid hormones. Endocrinology (1999) 140(4):1567–74. 10.1210/endo.140.4.6617 10098489

[30] Machinal-Quelin F Dieudonne MN Pecquery R Leneveu MC Giudicelli Y. Direct in vitro effects of androgens and estrogens on ob gene expression and leptin secretion in human adipose tissue. Endocrine (2002) 18(2):179–84. 10.1385/ENDO:18:2:179 12374466

[31] Wabitsch M Blum WF Muche R Braun M Hube F Rascher W Contribution of androgens to the gender difference in leptin production in obese children and adolescents. J Clin Invest (1997) 100(4):808–13. 10.1172/JCI119595 9259579 PMC508252

[32] Luukkaa V Pesonen U Huhtaniemi I Lehtonen A Tilvis R Tuomilehto J Inverse correlation between serum testosterone and leptin in men. J Clin Endocrinol Metabol. (1998) 83(9):3243–6. 10.1210/jcem.83.9.5134 9745436

[33] Zhang Y Olbort M Schwarzer K Nuesslein-Hildesheim B Nicolson M Murphy E The leptin receptor mediates apparent autocrine regulation of leptin gene expression. Biochem Biophys Res Commun (1997) 240(2):492–5. 10.1006/bbrc.1997.7622 9388507

[34] Colli S Silveira Cavalcante F Peixoto Martins M Sampaio FJ da Fonte Ramos C. Leptin role in the rat prostate ventral lobe. Fertil Steril (2011) 95(4):1490e–3e. 10.1016/j.fertnstert.2010.12.029 21257164

[35] Schwartz MW Seeley RJ Campfield LA Burn P Baskin DG. Identification of targets of leptin action in rat hypothalamus. J Clin Invest (1996) 98(5):1101–6. 10.1172/JCI118891 8787671 PMC507530

[36] Korner J Savontaus E Chua SC Jr. Leibel RL Wardlaw SL. Leptin regulation of Agrp and Npy mRNA in the rat hypothalamus. J Neuroendocrinol (2001) 13(11):959–66. 10.1046/j.1365-2826.2001.00716.x 11737554

[37] Swart I Jahng JW Overton JM Houpt TA. Hypothalamic NPY, AGRP, and POMC mRNA responses to leptin and refeeding in mice. Am J Physiol Regul Integr Comp Physiol (2002) 283(5):R1020–6. 10.1152/ajpregu.00501.2001 12376393

[38] Munzberg H Huo L Nillni EA Hollenberg AN Bjorbaek C. Role of signal transducer and activator of transcription 3 in regulation of hypothalamic proopiomelanocortin gene expression by leptin. Endocrinology (2003) 144(5):2121–31. 10.1210/en.2002-221037 12697721

[39] Magni P Vettor R Pagano C Calcagno A Beretta E Messi E Expression of a leptin receptor in immortalized gonadotropin-releasing hormone-secreting neurons. Endocrinology (1999) 140(4):1581–5. 10.1210/endo.140.4.6622 10098491

[40] Lebrethon MC Vandersmissen E Gerard A Parent AS Junien JL Bourguignon JP. In vitro stimulation of the prepubertal rat gonadotropin-releasing hormone pulse generator by leptin and neuropeptide Y through distinct mechanisms. Endocrinology (2000) 141(4):1464–9. 10.1210/endo.141.4.7432 10746651

[41] Watanobe H. Leptin directly acts within the hypothalamus to stimulate gonadotropin-releasing hormone secretion in vivo in rats. J Physiol (2002) 545(Pt 1):255–68. 10.1113/jphysiol.2002.023895 12433965 PMC2290656

[42] Lantos TA Gorcs TJ Palkovits M. Immunohistochemical mapping of neuropeptides in the premamillary region of the hypothalamus in rats. Brain Res Brain Res Rev (1995) 20(2):209–49. 10.1016/0165-0173(94)00013-F 7795657

[43] Cunningham MJ Clifton DK Steiner RA. Leptin’s actions on the reproductive axis: perspectives and mechanisms. Biol Reprod (1999) 60(2):216–22. 10.1095/biolreprod60.2.216 9915984

[44] Quennell JH Mulligan AC Tups A Liu X Phipps SJ Kemp CJ Leptin indirectly regulates gonadotropin-releasing hormone neuronal function. Endocrinology (2009) 150(6):2805–12. 10.1210/en.2008-1693 19179437 PMC2732287

[45] Louis GW Greenwald-Yarnell M Phillips R Coolen LM Lehman MN Myers MG Jr. Molecular mapping of the neural pathways linking leptin to the neuroendocrine reproductive axis. Endocrinology (2011) 152(6):2302–10. 10.1210/en.2011-0096 21427219 PMC3100610

[46] Caprio M Isidori AM Carta AR Moretti C Dufau ML Fabbri A. Expression of functional leptin receptors in rodent Leydig cells. Endocrinology (1999) 140(11):4939–47. 10.1210/en.140.11.4939 10537117

[47] Hu E Liang P Spiegelman BM. AdipoQ is a novel adipose-specific gene dysregulated in obesity. J Biol Chem (1996) 271(18):10697–703. 10.1074/jbc.271.18.10697 8631877

[48] Lu M Tang Q Olefsky JM Mellon PL Webster NJ. Adiponectin activates adenosine monophosphate-activated protein kinase and decreases luteinizing hormone secretion in LbetaT2 gonadotropes. Mol Endocrinol (2008) 22(3):760–71. 10.1210/me.2007-0330 18006641 PMC2262174

[49] Rodriguez-Pacheco F Martinez-Fuentes AJ Tovar S Pinilla L Tena-Sempere M Dieguez C Regulation of pituitary cell function by adiponectin. Endocrinology (2007) 148(1):401–10. 10.1210/en.2006-1019 17038552

[50] Cheng XB Wen JP Yang J Yang Y Ning G Li XY. GnRH secretion is inhibited by adiponectin through activation of AMP-activated protein kinase and extracellular signal-regulated kinase. Endocrine (2011) 39(1):6–12. 10.1007/s12020-010-9375-8 21052866

[51] Kim J Zheng W Grafer C Mann ML Halvorson LM. GnRH decreases adiponectin expression in pituitary gonadotropes via the calcium and PKA pathways. Reprod Sci (2013) 20(8):937–45. 10.1177/1933719112468947 23239819

[52] Caminos JE Nogueiras R Gaytan F Pineda R Gonzalez CR Barreiro ML Novel expression and direct effects of adiponectin in the rat testis. Endocrinology (2008) 149(7):3390–402. 10.1210/en.2007-1582 18403483

[53] Chabrolle C Tosca L Rame C Lecomte P Royere D Dupont J. Adiponectin increases insulin-like growth factor I-induced progesterone and estradiol secretion in human granulosa cells. Fertil Steril (2009) 92(6):1988–96. 10.1016/j.fertnstert.2008.09.008 19081562

[54] Fernandez-Fernandez R Tena-Sempere M Navarro VM Barreiro ML Castellano JM Aguilar E Effects of ghrelin upon gonadotropin- releasing hormone and gonadotropin secretion in adult female rats: in vivo and in vitro studies. Neuroendocrinology (2005) 82(5–6):245–55. 10.1159/000092753 16721030

[55] Khorram O Pau KY Spies HG. Release of hypothalamic neuropeptide Y and effects of exogenous NPY on the release of hypothalamic GnRH and pituitary gonadotropins in intact and ovariectomized does in vitro. Peptides (1988) 9(2):411–7. 10.1016/0196-9781(88)90277-X 3131748

[56] Sutton SW Toyama TT Otto S Plotsky PM. Evidence that neuropeptide Y (NPY) released into the hypophysial-portal circulation participates in priming gonadotropes to the effects of gonadotropin releasing hormone (GnRH). Endocrinology (1988) 123(2):1208–10. 10.1210/endo-123-2-1208 3293982

[57] Roa J Herbison AE. Direct regulation of GnRH neuron excitability by arcuate nucleus POMC and NPY neuron neuropeptides in female mice. Endocrinology (2012) 153(11):5587–99. 10.1210/en.2012-1470 22948210

[58] Israel DD Sheffer-Babila S de Luca C Jo YH Liu SM Xia Q Effects of leptin and melanocortin signaling interactions on pubertal development and reproduction. Endocrinology (2012) 153(5):2408–19. 10.1210/en.2011-1822 22408174 PMC3381095

[59] Sheffer-Babila S Sun Y Israel DD Liu SM Neal-Perry G Chua SC Jr. Agouti-related peptide plays a critical role in leptin’s effects on female puberty and reproduction. Am J Physiol Endocrinol Metabol. (2013) 305(12):E1512–20. 10.1152/ajpendo.00241.2013 24169048 PMC3882375

[60] Wu Q Whiddon BB Palmiter RD Ablation of neurons expressing agouti-related protein, but not melanin concentrating hormone, in leptin-deficient mice restores metabolic functions and fertility. Proc Natl Acad Sci U S A (2012) 109(8):3155–60 10.1073/pnas.1120501109 22232663 PMC3286929

[61] Lebrethon MC Vandersmissen E Gerard A Parent AS Bourguignon JP Cocaine and amphetamine-regulated-transcript peptide mediation of leptin stimulatory effect on the rat gonadotropin-releasing hormone pulse generator in vitro. J Neuroendocrinol (2000) 12(5):383–5 10.1046/j.1365-2826.2000.00497.x 10792575

[62] Parent AS Lebrethon MC Gerard A Vandersmissen E Bourguignon JP. Leptin effects on pulsatile gonadotropin releasing hormone secretion from the adult rat hypothalamus and interaction with cocaine and amphetamine regulated transcript peptide and neuropeptide Y. Regul Pept (2000) 92(1–3):17–24. 10.1016/S0167-0115(00)00144-0 11024560

[63] Popa SM Moriyama RM Caligioni CS Yang JJ Cho CM Concepcion TL Redundancy in Kiss1 expression safeguards reproduction in the mouse. Endocrinology (2013) 154(8):2784–94. 10.1210/en.2013-1222 23736293 PMC3713212

[64] Donato J Jr. Cravo RM Frazao R Gautron L Scott MM Lachey J Leptin’s effect on puberty in mice is relayed by the ventral premammillary nucleus and does not require signaling in Kiss1 neurons. J Clin Invest (2011) 121(1):355–68. 10.1172/JCI45106 21183787 PMC3007164

[65] Mayer C Boehm U. Female reproductive maturation in the absence of kisspeptin/GPR54 signaling. Nat Neurosci (2011) 14(6):704–10. 10.1038/nn.2818 21516099

[66] Cravo RM Frazao R Perello M Osborne-Lawrence S Williams KW Zigman JM Leptin signaling in Kiss1 neurons arises after pubertal development. PLoS One (2013) 8(3):e58698 10.1371/journal.pone.0058698 23505551 PMC3591417

[67] Backholer K Smith JT Rao A Pereira A Iqbal J Ogawa S Kisspeptin cells in the ewe brain respond to leptin and communicate with neuropeptide Y and proopiomelanocortin cells. Endocrinology (2010) 151(5):2233–43. 10.1210/en.2009-1190 20207832

[68] Qiu X Dowling AR Marino JS Faulkner LD Bryant B Bruning JC Delayed puberty but normal fertility in mice with selective deletion of insulin receptors from Kiss1 cells. Endocrinology (2013) 154(3):1337–48. 10.1210/en.2012-2056 23392256 PMC3578993

[69] Caprio M Fabbrini E Ricci G Basciani S Gnessi L Arizzi M Ontogenesis of leptin receptor in rat Leydig cells. Biol Reprod (2003) 68(4):1199–207. 10.1095/biolreprod.102.007831 12606446

[70] Tena-Sempere M Pinilla L Zhang FP Gonzalez LC Huhtaniemi I Casanueva FF Developmental and hormonal regulation of leptin receptor (Ob-R) messenger ribonucleic acid expression in rat testis. Biol Reprod (2001) 64(2):634–43. 10.1095/biolreprod64.2.634 11159367

[71] Herrid M O’Shea T McFarlane JR. Ontogeny of leptin and its receptor expression in mouse testis during the postnatal period. Mol Reprod Dev (2008) 75(5):874–80. 10.1002/mrd.20796 17935159

[72] El-Hefnawy T Ioffe S Dym M. Expression of the leptin receptor during germ cell development in the mouse testis. Endocrinology (2000) 141(7):2624–30. 10.1210/endo.141.7.7542 10875267

[73] Yuan M Huang G Li J Zhang J Li F Li K Hyperleptinemia directly affects testicular maturation at different sexual stages in mice, and suppressor of cytokine signaling 3 is involved in this process. Reprod Biol Endocrinol (2014) 12:15. 10.1186/1477-7827-12-15 24502529 PMC4015707

[74] Jones N Harrison GA Genetically determined obesity and sterility in the mouse. Proc Soc Study Fertil (1957) 9:51–64. 13580715

[75] Swerdloff RS Batt RA Bray GA. Reproductive hormonal function in the genetically obese (ob/ob) mouse. Endocrinology (1976) 98(6):1359–64. 10.1210/endo-98-6-1359 1278106

[76] Barash IA Cheung CC Weigle DS Ren H Kabigting EB Kuijper JL Leptin is a metabolic signal to the reproductive system. Endocrinology (1996) 137(7):3144–7. 10.1210/endo.137.7.8770941 8770941

[77] Banks WA McLay RN Kastin AJ Sarmiento U Scully S. Passage of leptin across the blood-testis barrier. Am J Physiol (1999) 276(6 Pt 1):E1099–104. 10362623 10.1152/ajpendo.1999.276.6.E1099

[78] von Sobbe HU Koebnick C Jenne L Kiesewetter F. Leptin concentrations in semen are correlated with serum leptin and elevated in hypergonadotrophic hypogonadism. Andrologia (2003) 35(4):233–7. 10.1046/j.1439-0272.2003.00565.x 12950408

[79] Fui MN Dupuis P Grossmann M. Lowered testosterone in male obesity: mechanisms, morbidity and management. Asian J Androl (2014) 16(2):223–31. 10.4103/1008-682X.122365 24407187 PMC3955331

[80] Isidori AM Caprio M Strollo F Moretti C Frajese G Isidori A Leptin and androgens in male obesity: evidence for leptin contribution to reduced androgen levels. J Clin Endocrinol Metab (1999) 84(10):3673–80. 10.1210/jcem.84.10.6082 10523013

[81] Tena-Sempere M Manna PR Zhang FP Pinilla L González LC Diéguez C Molecular mechanisms of leptin action in adult rat testis: potential targets for leptin-induced inhibition of steroidogenesis and pattern of leptin receptor messenger ribonucleic acid expression. J Endocrinol (2001) 170(2):413–23. 10.1677/joe.0.1700413 11479137

[82] Kruse M Bornstein SR Uhlmann K Paeth G Scherbaum WA. Leptin down-regulates the steroid producing system in the adrenal. Endocr Res (1998) 24(3–4):587–90. 10.3109/07435809809032650 9888542

[83] Cherradi N Capponi AM Gaillard RC Pralong FP. Decreased expression of steroidogenic acute regulatory protein: a novel mechanism participating in the leptin-induced inhibition of glucocorticoid biosynthesis. Endocrinology (2001) 142(8):3302–8. 10.1210/endo.142.8.8341 11459771

[84] Serke H Nowicki M Kosacka J Schroder T Kloting N Bluher M Leptin-deficient (ob/ob) mouse ovaries show fatty degeneration, enhanced apoptosis and decreased expression of steroidogenic acute regulatory enzyme. Int J Obes (2012) 36(8):1047–53. 10.1038/ijo.2011.220 22083551

[85] Subbaramaiah K Howe LR Bhardwaj P Du B Gravaghi C Yantiss RK Obesity is associated with inflammation and elevated aromatase expression in the mouse mammary gland. Cancer Prev Res (2011) 4(3):329–46. 10.1158/1940-6207.CAPR-10-0381 21372033 PMC3071249

[86] Liu E Samad F Mueller BM. Local adipocytes enable estrogen-dependent breast cancer growth: role of leptin and aromatase. Adipocyte. (2013) 2(3):165–9. 10.4161/adip.23645 23991363 PMC3756105

[87] Camina JP Lage M Menendez C Grana M Garcia-Devesa J Dieguez C Evidence of free leptin in human seminal plasma. Endocrine (2002) 17(3):169–74. 10.1385/ENDO:17:3:169 12108516

[88] Glander HJ Lammert A Paasch U Glasow A Kratzsch J. Leptin exists in tubuli seminiferi and in seminal plasma. Andrologia (2002) 34(4):227–33. 10.1046/j.1439-0272.2002.00501.x 12220230

[89] Jope T Lammert A Kratzsch J Paasch U Glander HJ. Leptin and leptin receptor in human seminal plasma and in human spermatozoa. Int J Androl (2003) 26(6):335–41. 10.1111/j.1365-2605.2003.00434.x 14636218

[90] Dandona P Dhindsa S. Update: hypogonadotropic hypogonadism in type 2 diabetes and obesity. J Clin Endocrinol Metab (2011) 96(9):2643–51. 10.1210/jc.2010-2724 21896895 PMC3167667

[91] Hofstra J Loves S van Wageningen B Ruinemans-Koerts J Jansen I de Boer H. High prevalence of hypogonadotropic hypogonadism in men referred for obesity treatment. Neth J Med (2008) 66(3):103–9. 18349465

[92] Esposito K Giugliano D Obesity, the metabolic syndrome, and sexual dysfunction in men. Clin Pharmacol Ther (2011) 90(1):169–73 10.1038/clpt.2011.91 21613988

[93] Zhao J Zhai L Liu Z Wu S Xu L. Leptin level and oxidative stress contribute to obesity-induced low testosterone in murine testicular tissue. Oxid Med Cell Longev. (2014) 2014:190945. 10.1155/2014/190945 24829619 PMC4009340

[94] Turner TT Lysiak JJ. Oxidative stress: a common factor in testicular dysfunction. J Androl (2008) 29(5):488–98. 10.2164/jandrol.108.005132 18567643

[95] De Liz Oliveira Cavalli VL Cattani D Heinz Rieg CE Pierozan P Zanatta L Parisotto EB Roundup disrupts male reproductive functions by triggering calcium-mediated cell death in rat testis and Sertoli cells. Free Radical Biol Med (2013) 65:335–46. 10.1016/j.freeradbiomed.2013.06.043 23820267

[96] Zamoner A Barreto KP Filho DW Sell F Woehl VM Rodrigues Guma FC Propylthiouracil-induced congenital hypothyroidism upregulates vimentin phosphorylation and depletes antioxidant defenses in immature rat testis. J Mol Endocrinol (2008) 40(3–4):125–35. 10.1677/JME-07-0089 18316471

[97] Zamoner A Barreto KP Filho DW Sell F Woehl VM Rodrigues Guma FC. Hyperthyroidism in the developing rat testis is associated with oxidative stress and hyperphosphorylated vimentin accumulation. Mol Cell Endocrinol (2007) 267(1–2):116–26. 10.1016/j.mce.2007.01.005 17306450

[98] Stokes VJ Anderson RA George JT. How does obesity affect fertility in men – and what are the treatment options? Clin Endocrinol (2014). 10.1111/cen.12591 25138694

[99] Rago V Aquila S Guido C Carpino A. Leptin and its receptor are expressed in the testis and in the epididymis of young and adult pigs. Anat Rec (2009) 292(5):736–45. 10.1002/ar.20880 19306434

[100] Lechan RM Fekete C. The TRH neuron: a hypothalamic integrator of energy metabolism. Prog Brain Res (2006) 153:209–35. 10.1016/S0079-6123(06)53012-2 16876577

[101] Kakucska I Rand W Lechan RM Thyrotropin-releasing hormone gene expression in the hypothalamic paraventricular nucleus is dependent upon feedback regulation by both triiodothyronine and thyroxine. Endocrinology (1992) 130(5):2845–50 10.1210/endo.130.5.1572297 1572297

[102] Segerson TP Kauer J Wolfe HC Mobtaker H Wu P Jackson IM Thyroid hormone regulates TRH biosynthesis in the paraventricular nucleus of the rat hypothalamus. Science (1987) 238(4823):78–80. 10.1126/science.3116669 3116669

[103] Tapia-Arancibia L Arancibia S Astier H. Evidence for alpha 1-adrenergic stimulatory control of in vitro release of immunoreactive thyrotropin-releasing hormone from rat median eminence: in vivo corroboration. Endocrinology (1985) 116(6):2314–9. 10.1210/endo-116-6-2314 2986944

[104] Fekete C Legradi G Mihaly E Huang QH Tatro JB Rand WM alpha-Melanocyte-stimulating hormone is contained in nerve terminals innervating thyrotropin-releasing hormone-synthesizing neurons in the hypothalamic paraventricular nucleus and prevents fasting-induced suppression of prothyrotropin-releasing hormone gene expression. J Neurosci (2000) 20(4):1550–8. 10662844 10.1523/JNEUROSCI.20-04-01550.2000 PMC6772359

[105] Fekete C Mihaly E Luo LG Kelly J Clausen JT Mao Q Association of cocaine- and amphetamine-regulated transcript-immunoreactive elements with thyrotropin-releasing hormone-synthesizing neurons in the hypothalamic paraventricular nucleus and its role in the regulation of the hypothalamic–pituitary–thyroid axis during fasting. J Neurosci (2000) 20(24):9224–34. 11125000 10.1523/JNEUROSCI.20-24-09224.2000 PMC6772999

[106] Harris M Aschkenasi C Elias CF Chandrankunnel A Nillni EA Bjoorbaek C Transcriptional regulation of the thyrotropin-releasing hormone gene by leptin and melanocortin signaling. J Clin Invest (2001) 107(1):111–20. 10.1172/JCI10741 11134186 PMC198547

[107] Perello M Stuart RC Nillni EA. The role of intracerebroventricular administration of leptin in the stimulation of prothyrotropin releasing hormone neurons in the hypothalamic paraventricular nucleus. Endocrinology (2006) 147(7):3296–306. 10.1210/en.2005-1533 16627588

[108] Toni R Jackson IM Lechan RM. Neuropeptide-Y-immunoreactive innervation of thyrotropin-releasing hormone-synthesizing neurons in the rat hypothalamic paraventricular nucleus. Endocrinology (1990) 126(5):2444–53. 10.1210/endo-126-5-2444 2109687

[109] Sawchenko PE Swanson LW Grzanna R Howe PR Bloom SR Polak JM. Colocalization of neuropeptide Y immunoreactivity in brainstem catecholaminergic neurons that project to the paraventricular nucleus of the hypothalamus. J Comp Neurol (1985) 241(2):138–53. 10.1002/cne.902410203 3840810

[110] Shioda S Nakai Y Sato A Sunayama S Shimoda Y. Electron-microscopic cytochemistry of the catecholaminergic innervation of TRH neurons in the rat hypothalamus. Cell Tissue Res (1986) 245(2):247–52. 10.1007/BF00213928 3091249

[111] ter Horst GJ Luiten PG. The projections of the dorsomedial hypothalamic nucleus in the rat. Brain Res Bull (1986) 16(2):231–48. 10.1016/0361-9230(86)90038-9 3697791

[112] Zamoner A Pessoa-Pureur R Mena Barreto Silva FR. Membrane-initiated actions of thyroid hormones on the male reproductive system. Life Sci (2011) 89(15–16):507–14. 10.1016/j.lfs.2011.04.006 21557952

[113] Zamoner A Pessoa-Pureur R Nongenomic actions of thyroid hormones: every why has a wherefore. Immunol. Endocr. Metabol. Agents Med. Chem. (2011) 11:165–78 10.2174/187152211796642765

[114] Gereben B Zavacki AM Ribich S Kim BW Huang SA Simonides WS Cellular and molecular basis of deiodinase-regulated thyroid hormone signaling. Endocr Rev (2008) 29(7):898–938. 10.1210/er.2008-0019 18815314 PMC2647704

[115] Bates JM St Germain DL Galton VA. Expression profiles of the three iodothyronine deiodinases, D1, D2, and D3, in the developing rat. Endocrinology (1999) 140(2):844–51. 10.1210/en.140.2.844 9927314

[116] Wajner SM dos Santos Wagner M Melo RC Parreira GG Chiarini-Garcia H Bianco AC Type 2 iodothyronine deiodinase is highly expressed in germ cells of adult rat testis. J Endocrinol (2007) 194(1):47–54. 10.1677/JOE-07-0106 17592020

[117] Visser WE Friesema EC Jansen J Visser TJ Thyroid hormone transport in and out of cells. Trends Endocrinol Metab (2008) 19(2):50–6 10.1016/j.tem.2007.11.003 18291666

[118] Sande S Privalsky ML. Identification of TRACs (T3 receptor-associating cofactors), a family of cofactors that associate with, and modulate the activity of, nuclear hormone receptors. Mol Endocrinol (1996) 10(7):813–25. 10.1210/mend.10.7.8813722 8813722

[119] Zamoner A Pessoa-Pureur R Silva FR. Membrane-initiated actions of thyroid hormones on the male reproductive system. Life Sci (2011) 89(15–16):507–14. 10.1016/j.lfs.2011.04.006 21557952

[120] Tsai MJ O’Malley BW Molecular mechanisms of action of steroid/thyroid receptor superfamily members. Annu Rev Biochem (1994) 63:451–86 10.1146/annurev.bi.63.070194.002315 7979245

[121] Yen PM Ando S Feng X Liu Y Maruvada P Xia X. Thyroid hormone action at the cellular, genomic and target gene levels. Mol Cell Endocrinol (2006) 246(1–2):121–7. 10.1016/j.mce.2005.11.030 16442701

[122] Patrick L. Thyroid disruption: mechanism and clinical implications in human health. Altern Med Rev (2009) 14(4):326–46. 20030460

[123] Baniahmad A Kohne AC Renkawitz R. A transferable silencing domain is present in the thyroid hormone receptor, in the v-erbA oncogene product and in the retinoic acid receptor. EMBO J (1992) 11(3):1015–23. 1347744 10.1002/j.1460-2075.1992.tb05140.x PMC556542

[124] Brent GA Dunn MK Harney JW Gulick T Larsen PR Moore DD. Thyroid hormone aporeceptor represses T3-inducible promoters and blocks activity of the retinoic acid receptor. New Biol (1989) 1(3):329–36. 2562125

[125] Damm K Thompson CC Evans RM. Protein encoded by v-erbA functions as a thyroid-hormone receptor antagonist. Nature (1989) 339(6226):593–7. 10.1038/339593a0 2733791

[126] Fondell JD Brunel F Hisatake K Roeder RG. Unliganded thyroid hormone receptor alpha can target TATA-binding protein for transcriptional repression. Mol Cell Biol (1996) 16(1):281–7. 8524305 10.1128/mcb.16.1.281 PMC231001

[127] Graupner G Wills KN Tzukerman M Zhang XK Pfahl M. Dual regulatory role for thyroid-hormone receptors allows control of retinoic-acid receptor activity. Nature (1989) 340(6235):653–6. 10.1038/340653a0 2549424

[128] Sap J Munoz A Schmitt J Stunnenberg H Vennstrom B. Repression of transcription mediated at a thyroid hormone response element by the v-erb-A oncogene product. Nature (1989) 340(6230):242–4. 10.1038/340242a0 2569164

[129] Arambepola NK Bunick D Cooke PS. Thyroid hormone effects on androgen receptor messenger RNA expression in rat Sertoli and peritubular cells. J Endocrinol (1998) 156(1):43–50. 10.1677/joe.0.1560043 9496232

[130] Buzzard JJ Morrison JR O’Bryan MK Song Q Wreford NG. Developmental expression of thyroid hormone receptors in the rat testis. Biol Reprod (2000) 62(3):664–9. 10.1095/biolreprod62.3.664 10684808

[131] Canale D Agostini M Giorgilli G Caglieresi C Scartabelli G Nardini V Thyroid hormone receptors in neonatal, prepubertal, and adult rat testis. J Androl (2001) 22(2):284–8. 11229803

[132] Rao JN Liang JY Chakraborti P Feng P. Effect of thyroid hormone on the development and gene expression of hormone receptors in rat testes in vivo. J Endocrinol Invest (2003) 26(5):435–43. 10.1007/BF03345199 12906371

[133] Jannini EA Crescenzi A Rucci N Screponi E Carosa E de Matteis A Ontogenetic pattern of thyroid hormone receptor expression in the human testis. J Clin Endocrinol Metab (2000) 85(9):3453–7. 10.1210/jcem.85.9.6803 10999848

[134] Nakai A Sakurai A Bell GI DeGroot LJ. Characterization of a third human thyroid hormone receptor coexpressed with other thyroid hormone receptors in several tissues. Mol Endocrinol (1988) 2(11):1087–92. 10.1210/mend-2-11-1087 2464749

[135] Zanatta AP Zanatta L Gonçalves R Zamoner A Silva FR. Rapid responses to reverse T_3_ hormone in immature rat Sertoli cells: calcium uptake and exocytosis mediated by integrin. PLoS One (2013) 8(10):e77176. 10.1371/journal.pone.0077176 24130850 PMC3795021

[136] Zanatta AP Zanatta L Goncalves R Zamoner A Mena Barreto Silva FR. Integrin participates in the effect of thyroxine on plasma membrane in immature rat testis. Biochim Biophys Acta (2013) 1830(3):2629–37. 10.1016/j.bbagen.2012.10.022 23137442

[137] Menegaz D Royer C Rosso A Pacheco de Souza AZ Soares dos Santos AR Mena Barreto Silva FR. Rapid stimulatory effect of thyroxine on plasma membrane transport systems: calcium uptake and neutral amino acid accumulation in immature rat testis. Int J Biochem Cell Biol (2010) 42(6):1046–51. 10.1016/j.biocel.2010.03.015 20348014

[138] Menegaz D Zamoner A Royer C Leite L Bortolottoc Z Silva F. Rapid responses to thyroxine in the testis: active protein synthesis-independent pathway. Mol Cell Endocrinol (2006) 246(1–2):128–34. 10.1016/j.mce.2005.11.019 16387420

[139] Mendis-Handagama SM Siril Ariyaratne HB. Leydig cells, thyroid hormones and steroidogenesis. Indian J Exp Biol (2005) 43(11):939–62. 16313060

[140] Fumel B Guerquin MJ Livera G Staub C Magistrini M Gauthier C Thyroid hormone limits postnatal Sertoli cell proliferation in vivo by activation of its alpha1 isoform receptor (TRalpha1) present in these cells and by regulation of Cdk4/JunD/c-myc mRNA levels in mice. Biol Reprod (2012) 87(1):16,1–9. 10.1095/biolreprod.111.098418 22539677

[141] Orth JM Gunsalus GL Lamperti AA. Evidence from Sertoli cell-depleted rats indicates that spermatid number in adults depends on numbers of Sertoli cells produced during perinatal development. Endocrinology (1988) 122(3):787–94. 10.1210/endo-122-3-787 3125042

[142] Tarulli GA Stanton PG Meachem SJ. Is the adult Sertoli cell terminally differentiated? Biol Reprod (2012) 87(1):1–11. 10.1095/biolreprod.111.095091 22492971

[143] Holsberger DR Buchold GM Leal MC Kiesewetter SE O’Brien DA Hess RA Cell-cycle inhibitors p27Kip1 and p21Cip1 regulate murine Sertoli cell proliferation. Biol Reprod (2005) 72(6):1429–36. 10.1095/biolreprod.105.040386 15728790

[144] Ulisse S Jannini EA Carosa E Piersanti D Graziano FM D’Armiento M. Inhibition of aromatase activity in rat Sertoli cells by thyroid hormone. J Endocrinol (1994) 140(3):431–6. 10.1677/joe.0.1400431 8182371

[145] Ando S Sirianni R Forastieri P Casaburi I Lanzino M Rago V Aromatase expression in prepuberal Sertoli cells: effect of thyroid hormone. Mol Cell Endocrinol (2001) 178(1–2):11–21. 10.1016/S0303-7207(01)00443-9 11403889

[146] Catalano S Pezzi V Chimento A Giordano C Carpino A Young M Triiodothyronine decreases the activity of the proximal promoter (PII) of the aromatase gene in the mouse Sertoli cell line, TM4. Mol Endocrinol (2003) 17(5):923–34. 10.1210/me.2002-0102 12586841

[147] Maran RR. Thyroid hormones: their role in testicular steroidogenesis. Arch Androl (2003) 49(5):375–88. 10.1080/01485010390204968 12893516

[148] Maran RR Arunakaran J Aruldhas MM. T3 directly stimulates basal and modulates LH induced testosterone and oestradiol production by rat Leydig cells in vitro. Endocr J (2000) 47(4):417–28. 10.1507/endocrj.47.417 11075722

[149] Romano RM Bargi-Souza P Brunetto EL Goulart-Silva F Avellar MC Oliveira CA Hypothyroidism in adult male rats alters posttranscriptional mechanisms of luteinizing hormone biosynthesis. Thyroid (2013) 23(4):497–505. 10.1089/thy.2011.0514 23240964

[150] Gilleron J Nebout M Scarabelli L Senegas-Balas F Palmero S Segretain D A potential novel mechanism involving connexin 43 gap junction for control of sertoli cell proliferation by thyroid hormones. J Cell Physiol (2006) 209(1):153–61. 10.1002/jcp.20716 16823880

[151] Levine N Marsh DJ. Micropuncture studies of the electrochemical aspects of fluid and electrolyte transport in individual seminiferous tubules, the epididymis and the vas deferens in rats. J Physiol (1971) 213(3):557–70. 5551402 10.1113/jphysiol.1971.sp009400 PMC1331741

[152] Jégou B The Sertoli cell in vivo and in vitro. Cell Biol Toxicol (1992) 8(3):49–54 10.1007/BF00130510 1446257

[153] Ko WH Chan HC Chew SB Wong PY. Regulated anion secretion in cultured epithelia from Sertoli cells of immature rats. J Physiol (1998) 512(Pt 2):471–80. 10.1111/j.1469-7793.1998.471be.x 9763636 PMC2231224

[154] Ko WH Au CL Yip CY. Multiple purinergic receptors lead to intracellular calcium increases in cultured rat Sertoli cells. Life Sci (2003) 72(13):1519–35. 10.1016/S0024-3205(02)02410-4 12535719

[155] Zanatta AP Zanatta L Goncalves R Zamoner A Mena Barreto Silva FR. Rapid responses to reverse T-3 hormone in immature rat sertoli cells: calcium uptake and exocytosis mediated by integrin. PLoS One (2013) 8(10):1–11. 10.1371/journal.pone.0077176 24130850 PMC3795021

[156] Tindall DJ Vitale R Means AR. Androgen binding protein as a biochemical marker of formation of the blood-testis barrier. Endocrinology (1975) 97(3):636–48. 10.1210/endo-97-3-636 1175511

[157] Carreau S Silandre D Bois C Bouraima H Galeraud-Denis I Delalande C. Estrogens: a new player in spermatogenesis. Folia Histochem Cytobiol (2007) 45(Suppl 1):S5–10. 18292817

[158] Papadopoulos V Carreau S Szerman-Joly E Drosdowsky MA Dehennin L Scholler R. Rat testis 17 beta-estradiol: identification by gas chromatography-mass spectrometry and age related cellular distribution. J Steroid Biochem (1986) 24(6):1211–6. 10.1016/0022-4731(86)90385-7 3736047

[159] Sisci D Panno ML Salerno M Maggiolini M Pezzi V Morrone EG A time course study on the “in vitro” effects of T3 and testosterone on androgen and estrogen receptors in peripuberal primary rat Sertoli cells. Exp Clin Endocrinol Diabetes (1997) 105(4):218–24. 10.1055/s-0029-1211755 9285209

[160] Pezzi V Panno ML Sirianni R Forastieri P Casaburi I Lanzino M Effects of tri-iodothyronine on alternative splicing events in the coding region of cytochrome P450 aromatase in immature rat Sertoli cells. J Endocrinol (2001) 170(2):381–93. 10.1677/joe.0.1700381 11479134

[161] Chiao YC Lee HY Wang SW Hwang JJ Chien CH Huang SW Regulation of thyroid hormones on the production of testosterone in rats. J Cell Biochem (1999) 73(4):554–62. 10.1002/(SICI)1097-4644(19990615)73:4<554::AID-JCB13>3.3.CO;2-C 10733348

[162] Manna PR Kero J Tena-Sempere M Pakarinen P Stocco DM Huhtaniemi IT. Assessment of mechanisms of thyroid hormone action in mouse Leydig cells: regulation of the steroidogenic acute regulatory protein, steroidogenesis, and luteinizing hormone receptor function. Endocrinology (2001) 142(1):319–31. 10.1210/endo.142.1.7900 11145595

[163] Manna PR Roy P Clark BJ Stocco DM Huhtaniemi IT. Interaction of thyroid hormone and steroidogenic acute regulatory (StAR) protein in the regulation of murine Leydig cell steroidogenesis. J Steroid Biochem Mol Biol (2001) 76(1–5):167–77. 10.1016/S0960-0760(00)00156-4 11384875

[164] Krassas GE Poppe K Glinoer D. Thyroid function and human reproductive health. Endocr Rev (2010) 31(5):702–55. 10.1210/er.2009-0041 20573783

[165] Iglesias P Diez JJ. Thyroid dysfunction and kidney disease. Eur J Endocrinol (2009) 160(4):503–15. 10.1530/EJE-08-0837 19095779

[166] Ascoli M Fanelli F Segaloff DL. The lutropin/choriogonadotropin receptor, a 2002 perspective. Endocr Rev (2002) 23(2):141–74. 10.1210/edrv.23.2.0462 11943741

[167] Ahima RS Flier JS. Leptin. Annu Rev Physiol (2000) 62:413–37. 10.1146/annurev.physiol.62.1.413 10845097

[168] Araujo RL Andrade BM da Silva ML Ferreira AC Carvalho DP. Tissue-specific deiodinase regulation during food restriction and low replacement dose of leptin in rats. Am J Physiol Endocrinol Metab (2009) 296(5):E1157–63. 10.1152/ajpendo.90869.2008 19208852

[169] Cabanelas A Lisboa PC Moura EG Pazos-Moura CC. Leptin acute modulation of the 5′-deiodinase activities in hypothalamus, pituitary and brown adipose tissue of fed rats. Horm Metab Res (2006) 38(8):481–5. 10.1055/s-2006-949527 16941271

[170] Ortiga-Carvalho TM Oliveira KJ Soares BA Pazos-Moura CC. The role of leptin in the regulation of TSH secretion in the fed state: in vivo and in vitro studies. J Endocrinol (2002) 174(1):121–5. 10.1677/joe.0.1740121 12098670

[171] Seoane LM Carro E Tovar S Casanueva FF Dieguez C. Regulation of in vivo TSH secretion by leptin. Regul Pept (2000) 92(1–3):25–9. 10.1016/S0167-0115(00)00145-2 11024561

[172] Elias CF Kelly JF Lee CE Ahima RS Drucker DJ Saper CB Chemical characterization of leptin-activated neurons in the rat brain. J Comp Neurol (2000) 423(2):261–81. 10.1002/1096-9861(20000724)423:2<261::AID-CNE6>3.0.CO;2-6 10867658

[173] Guo F Bakal K Minokoshi Y Hollenberg AN. Leptin signaling targets the thyrotropin-releasing hormone gene promoter in vivo. Endocrinology (2004) 145(5):2221–7. 10.1210/en.2003-1312 14764630

[174] Nillni EA Vaslet C Harris M Hollenberg A Bjorbak C Flier JS. Leptin regulates prothyrotropin-releasing hormone biosynthesis. Evidence for direct and indirect pathways. J Biol Chem (2000) 275(46):36124–33. 10.1074/jbc.M003549200 10967095

[175] Kim MS Small CJ Stanley SA Morgan DG Seal LJ Kong WM The central melanocortin system affects the hypothalamo-pituitary thyroid axis and may mediate the effect of leptin. J Clin Invest (2000) 105(7):1005–11. 10.1172/JCI8857 10749579 PMC377483

[176] Legradi G Emerson CH Ahima RS Flier JS Lechan RM. Leptin prevents fasting-induced suppression of prothyrotropin-releasing hormone messenger ribonucleic acid in neurons of the hypothalamic paraventricular nucleus. Endocrinology (1997) 138(6):2569–76. 10.1210/endo.138.6.5209 9165050

[177] Costa-e-Sousa RH Hollenberg AN. Minireview: the neural regulation of the hypothalamic–pituitary–thyroid axis. Endocrinology (2012) 153(9):4128–35. 10.1210/en.2012-1467 22759379 PMC3423621

[178] Nillni EA. Regulation of the hypothalamic thyrotropin releasing hormone (TRH) neuron by neuronal and peripheral inputs. Front Neuroendocrinol (2010) 31(2):134–56. 10.1016/j.yfrne.2010.01.001 20074584 PMC2849853

[179] Menendez C Baldelli R Camina JP Escudero B Peino R Dieguez C TSH stimulates leptin secretion by a direct effect on adipocytes. J Endocrinol (2003) 176(1):7–12. 10.1677/joe.0.1760007 12525244

[180] Casanueva FF Dieguez C. Neuroendocrine regulation and actions of leptin. Front Neuroendocrinol (1999) 20(4):317–63. 10.1006/frne.1999.0187 10569281

[181] Coiro V Volpi R Chiodera P. Mediation by nitric oxide of TRH-, but not metoclopramide-stimulated TSH secretion in humans. Neuroreport (1995) 6(8):1174–6. 10.1097/00001756-199505300-00025 7662901

[182] Radwanska P Kosior-Korzecka U. Effect of leptin on thyroid-stimulating hormone secretion and nitric oxide release from pituitary cells of ewe lambs in vitro. J Physiol Pharmacol (2014) 65(1):145–51. 24622839

[183] Mantzoros CS Flier JS Rogol AD. A longitudinal assessment of hormonal and physical alterations during normal puberty in boys. V. Rising leptin levels may signal the onset of puberty. J Clin Endocrinol Metabol. (1997) 82(4):1066–70. 10.1210/jcem.82.4.3878 9100574

[184] Carlsson B Ankarberg C Rosberg S Norjavaara E Albertsson-Wikland K Carlsson LM. Serum leptin concentrations in relation to pubertal development. Archi Dis Child. (1997) 77(5):396–400. 10.1136/adc.77.5.396 9487960 PMC1717396

[185] Mann DR Akinbami MA Gould KG Castracane VD. Leptin and thyroxine during sexual development in male monkeys: effect of neonatal gonadotropin-releasing hormone antagonist treatment and delayed puberty on the developmental pattern of leptin and thyroxine secretion. Eur J Endocrinol (2002) 146(6):891–8. 10.1530/eje.0.1460891 12039711

[186] Farooqi IS Matarese G Lord GM Keogh JM Lawrence E Agwu C Beneficial effects of leptin on obesity, T cell hyporesponsiveness, and neuroendocrine/metabolic dysfunction of human congenital leptin deficiency. J Clin Invest (2002) 110(8):1093–103. 10.1172/JCI0215693 12393845 PMC150795

[187] Gill MS Hall CM Tillmann V Clayton PE. Constitutional delay in growth and puberty (CDGP) is associated with hypoleptinaemia. Clin Endocrinol (Oxf). (1999) 50(6):721–6. 10.1046/j.1365-2265.1999.00736.x 10468943

[188] Sera N Yokoyama N Abe Y Ide A Imaizumi M Usa T Thyroid hormones influence serum leptin levels in patients with Graves’ disease during suppression of beta-adrenergic receptors. Thyroid (2000) 10(8):641–6. 10.1089/10507250050137707 11014307

[189] Asami T Ciomarten T Uchiyama M. Relationship between serum leptin and thyroid hormones in children. Pediatr Int (2000) 42(3):293–5. 10.1046/j.1442-200x.2000.01220.x 10881589

[190] Li R Hu Y Ni Y Xia D Grossmann R Zhao R. Leptin stimulates hepatic activation of thyroid hormones and promotes early posthatch growth in the chicken. Comp Biochem Physiol A Mol Integr Physiol (2011) 160(2):200–6. 10.1016/j.cbpa.2011.06.001 21679771

[191] Ortega FJ Jilkova ZM Moreno-Navarrete JM Pavelka S Rodriguez-Hermosa JI Kopeck Ygrave J Type I iodothyronine 5′-deiodinase mRNA and activity is increased in adipose tissue of obese subjects. Int J Obes (2012) 36(2):320–4. 10.1038/ijo.2011.101 21610697

[192] Calvino C Souza LL Costa-e-Sousa RH Almeida NA Trevenzoli IH Pazos-Moura CC. Hypothyroidism reduces ObRb-STAT3 leptin signalling in the hypothalamus and pituitary of rats associated with resistance to leptin acute anorectic action. J Endocrinol (2012) 215(1):129–35. 10.1530/JOE-11-0476 22875962

[193] Groba C Mayerl S van Mullem AA Visser TJ Darras VM Habenicht AJ Hypothyroidism compromises hypothalamic leptin signaling in mice. Mol Endocrinol (2013) 27(4):586–597. 10.1210/me.2012-1311 23518925 PMC5416808

